# Supplementary material for: Probing the link between vision and language in material perception using psychophysics and unsupervised learning
Source: PLoS Comput Biol. 2024 Oct 3;20(10):e1012481. doi: 10.1371/journal.pcbi.1012481 (PMC11478833; doi:10.1371/journal.pcbi.1012481)
Supplement: S1 Text — (PDF) [file pcbi.1012481.s001.pdf]

Supplementary Information

Probing the Link Between Vision and Language in Material Perception Using Psychophysics and Unsupervised Learning

Chenxi Liao[1*], Masataka Sawayama[3], and Bei Xiao[2]

1 American University, Department of Neuroscience, Washington DC, United States of America
2 American University, Department of Computer Science, Washington DC, United States of America
3 The University of Tokyo, Graduate School of Information Science and Technology, Tokyo, Japan
*cl6070a@american.edu

Psychophysical Stimuli.
We sampled 72 images from the Space of Morphable Material Appearance. The images were generated from our StyleGAN models. Top three rows are the images of 'Original' materials: Soap, Toy, and Rock. They were synthesized from the trained StyleGAN generators: G_{soap}, G_{toy}, and G_{rock}, respectively. The bottom three rows are the images of 'Morphed' materials: Soap-to-rock, Rock-to-toy, and Soap-to-toy. They were generated from the morphed StyleGAN generator: $G_{soap\text{-}to\text{-}rock}$, $G_{rock\text{-}to\text{-}toy}$, and $G_{soap\text{-}to\text{-}toy}$, respectively.

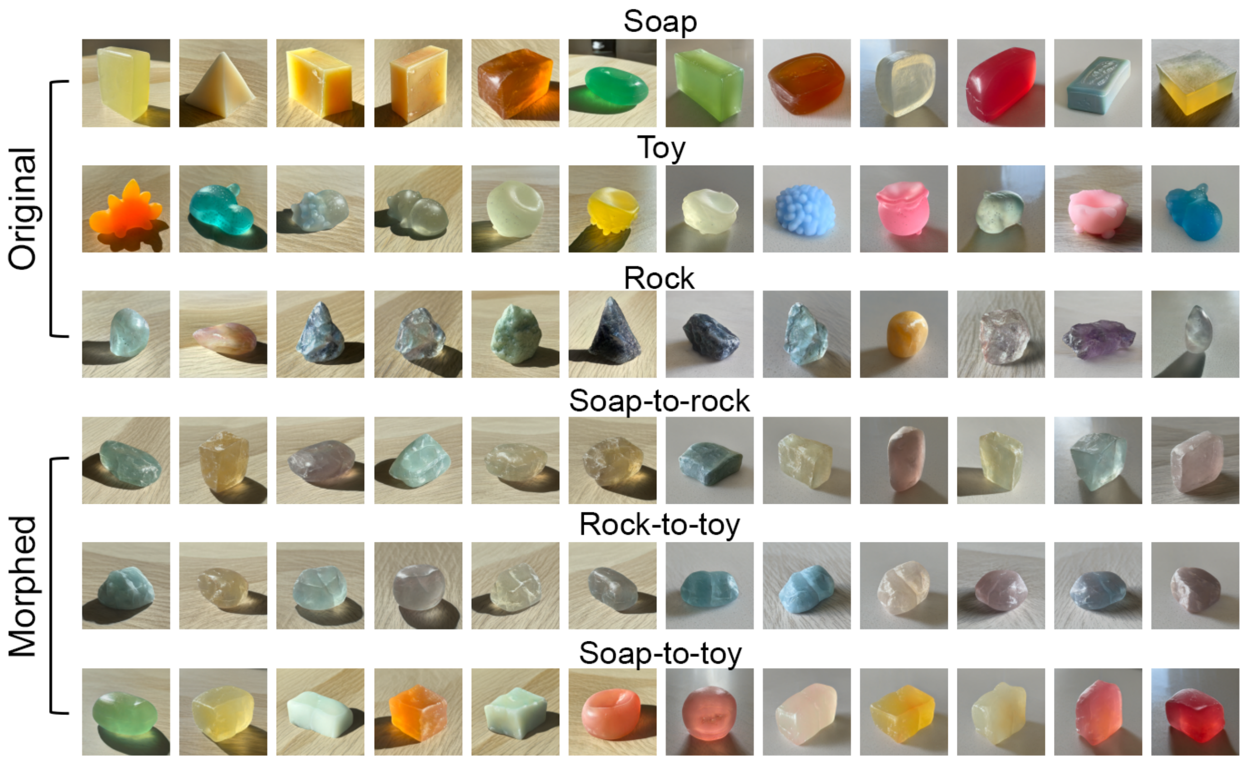

Fig S.1. Seventy-two generated images used as stimuli for both Multiple Arrangement and Verbal Description experiments.

Perceptual Representational Dissimilarity Matrices (RDMs) from psychophysical experiments.

Here, we present the individual participant's (N=16) Vision RDMs (from the Multiple Arrangement experiment) and Text RDMs (from the Verbal Description experiment).

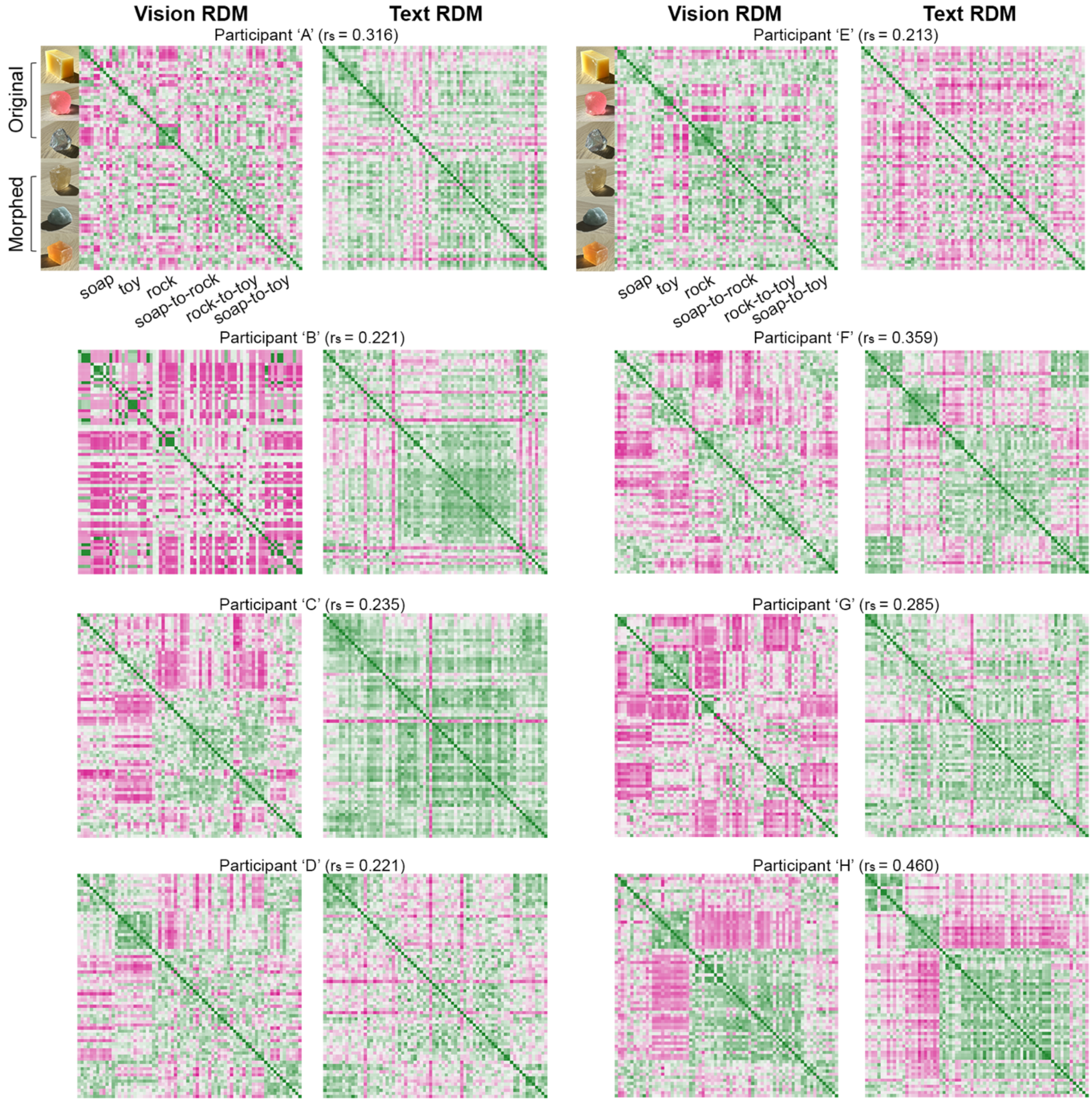

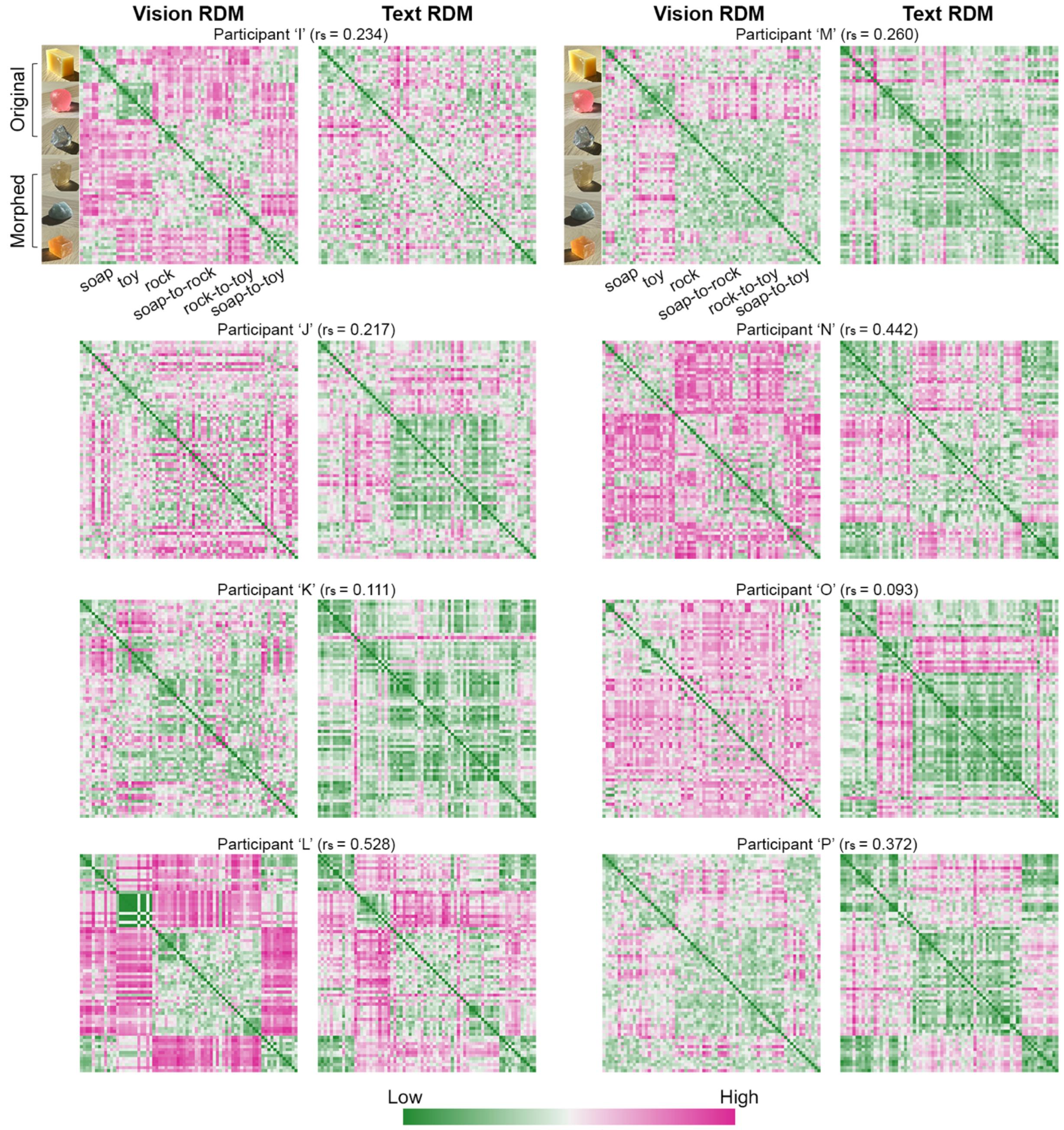

Fig S.2. Individual participant's (N=16) RDMs of visual material similarity judgment via Multiple Arrangement (Vision RDMs) and Verbal Description (Text RDMs). The Text RDMs are based on the CLIP's text embedding results, as illustrated in the main paper Fig 4A. The Spearman's correlation (r_s) between the participant's own Vision and Text RDMs is marked on top of each pair of RDMs.

Gromov-Wasserstein Optimal Transport (GWOT).
We applied the unsupervised alignment method GWOT to compare two similarity structures between the stimulus-level alignment between the Vision and Text RDMs. Here, we used OpenAI's Embedding V3-small to construct the individual participant's Text RDM and compute the group average Text RDM.

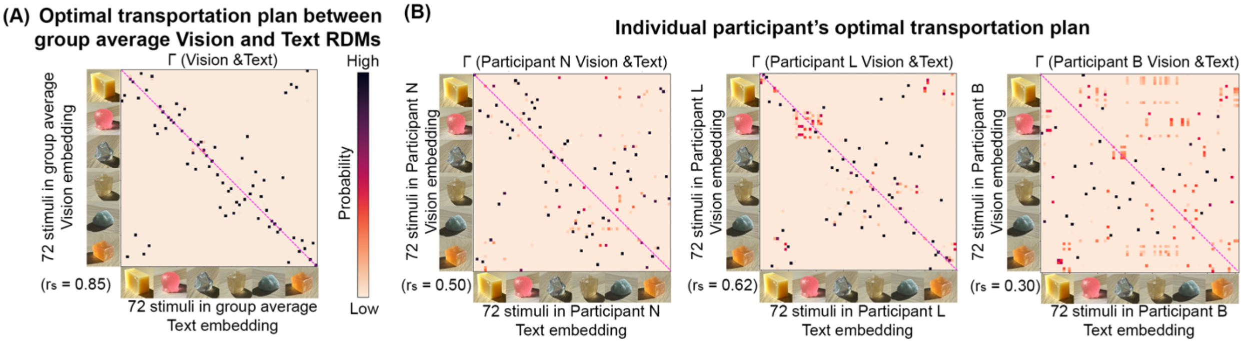

Fig S.3. Optimal transportation plan between Vision and Text RDMs. The Text RDMs are based on OpenAI's Embedding V3-small. (A) Optimal transportation plan matrix (Γ) between group average Vision and Text RDMs. (B) Optimal transportation plan matrix of individual participant's Vision and Text RDMs. The Spearman's correlation (r_s) between the Vision and Text RDMs is noted in the bottom left corner of the Γ matrix.

Effect of removing "material name" on vision-language correlation

We conducted additional analysis by comparing the Vision and Text RDMs (CLIP's embedding, or OpenAI Embedding V3-small) of the 36 images of 'original' (soap, rock, toy) and 36 images of morphed (soap-to-rock, soap-to-toy, toy-to-rock) materials. When 'material name' is removed from the word embedding, we observed a significant decrease in vision-language correlation for both 'original' and 'morphed' conditions (Wilcoxon one-sided signed-rank test, CLIP: original material $W(16) = 124$, $p = 0.001$, morphed material $W(16) = 119$, $p = 0.003$; OpenAI Embedding V3-small: original material $W(16) = 129$, $p = 0.0002$, morphed material $W(16) = 103$, $p = 0.037$).

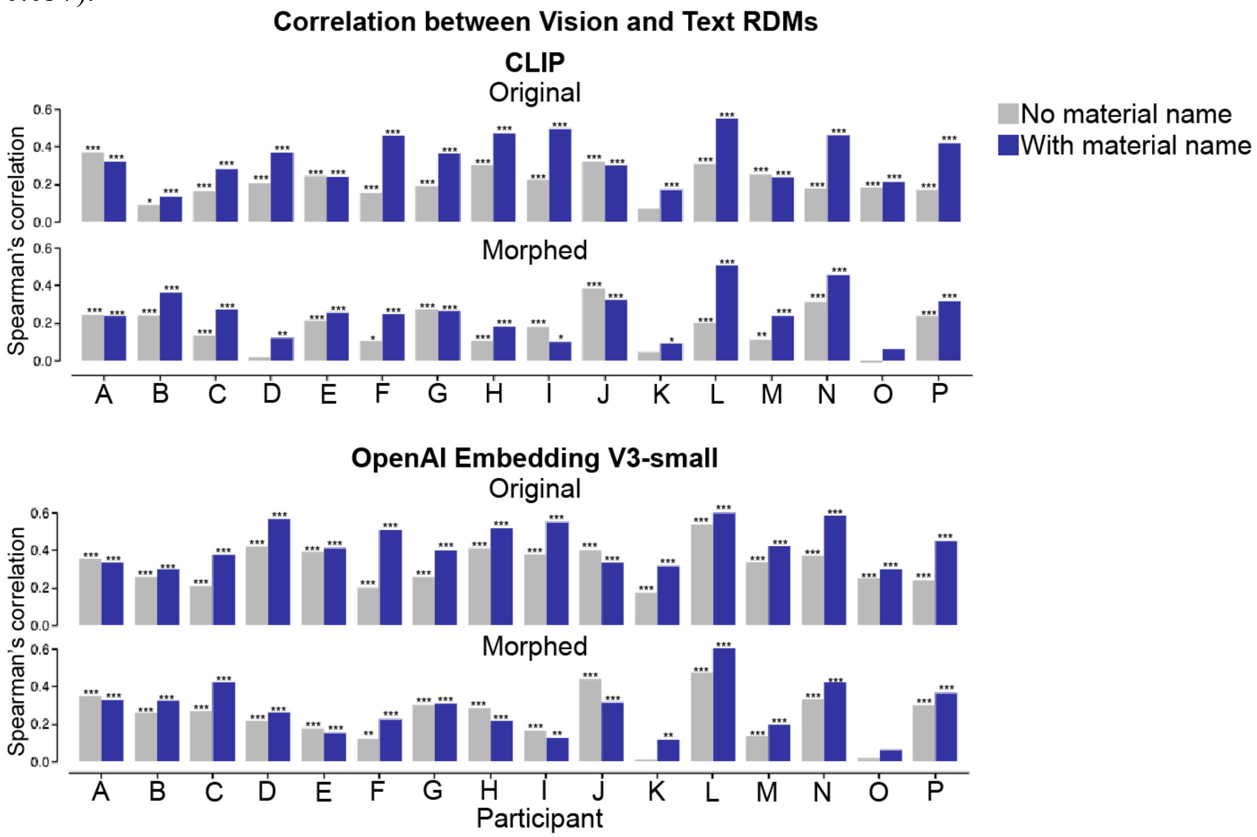

Fig S.4. Correlation between Vision and Text RDMs with ROI for the original "original" (soap, rock, toy) and "morphed" (soap-to-rock, soap-to-toy, toy-to-rock) materials. Top: text embedding derived from CLIP's text encoder. Bottom: text embedding derived from OpenAI Embedding V3-small. The blue bars indicate Spearman's correlation values when all text features are included to construct the Text RDM. The gray bars indicate the correlation values when the "material name" is excluded from constructing the Text RDM. Asterisks indicate FDR-corrected p-values: *** $p<0.001$, ** $p<0.01$, and * $p<0.05$.

Similar to the "With material name" condition, we computed the Group Average Text RDM by taking the mean of the individual participants' Text RDMs when the description of the material name is removed from the text feature embeddings (see Fig S.5A Leftmost panel). Hence, we applied the MDS to visualize the two-dimensional embedding of the "No material name" group average Text RDM. Compared with the MDS of the "With material name" (see Fig S.5B Middle panel), the data points in the "No material name" condition show less clear clustering (see Fig

S.5A Middle panel). When comparing group average Vision RDM and the Text RDM with GWOT, removing the material name results in an optimal transport plan matrix Γ (see Fig S.5A Rightmost panel) that has an even smaller fraction of diagonal elements in its diagonal in comparison to the Γ from "With material name" condition (see Fig S.5B Rightmost panel). The top-1 matching rate also significantly dropped (1.38%). This suggests that removing the material name may further reduce the one-to-one mapping between verbal description and visual judgment of stimuli.

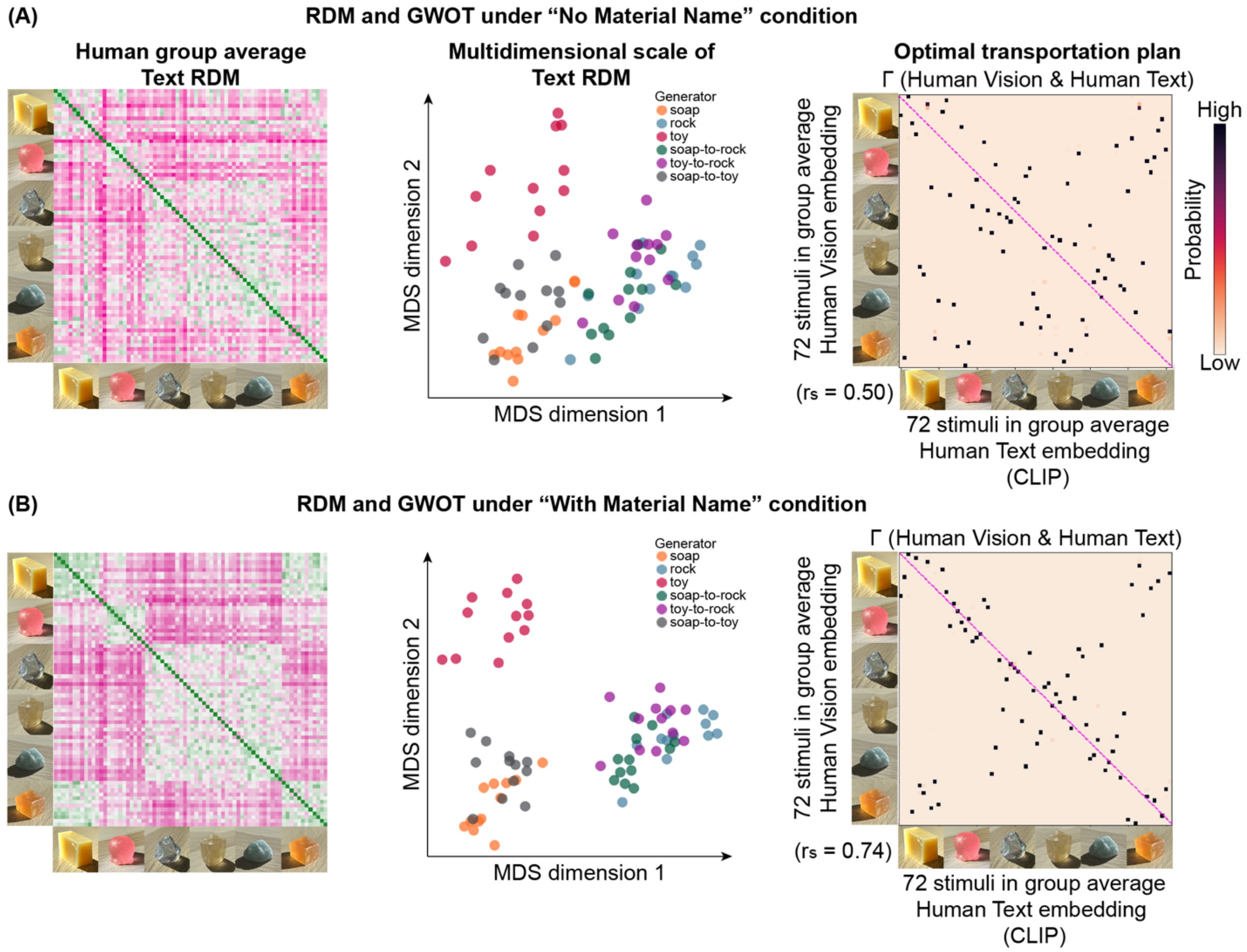

Fig S.5. Representational space of human text response in the "No Material Name" versus "With Material Name" condition based on CLIP's text embedding. Under each text embedding condition, the leftmost column shows the group average Text RDMs. The middle column is the MDS embeddings of the group average Text RDMs. The rightmost column shows the optimal transport plans that compare the group average human Vision RDM (from the Multiple Arrangement task) with the group average Text RDM. (A) "No Material Name" (when the "material name" is removed from the text embedding) (B) "With Material Name" condition.

Image representations from the pre-trained models.
We tested various visual-semantic models, self-supervised vision models, and perceptual similarity metrics. For the visual-semantic models, we examined the embeddings resulting from various versions (using either ResNet or Vision Transformer (ViT) as the backbone) of their image encoder: CLIP-ResNet50, CLIP-ViT-B/16, CLIP-ViT-B/32, CLIP-ViT-L/14, OpenCLIP-ViT-B/32, OpenCLIP-ViT-L/14, and OpenCLIP-ViT-H/14. For the self-supervised vision models, we tested the family of DINO: DINO (e.g., DINO-s8) and DINOV2 (DINOV2-small [1]). We evaluated the image similarity based on two established metrics: the lower-level patch-based metric LPIPS, and the more semantically aware metric DreamSim [2].

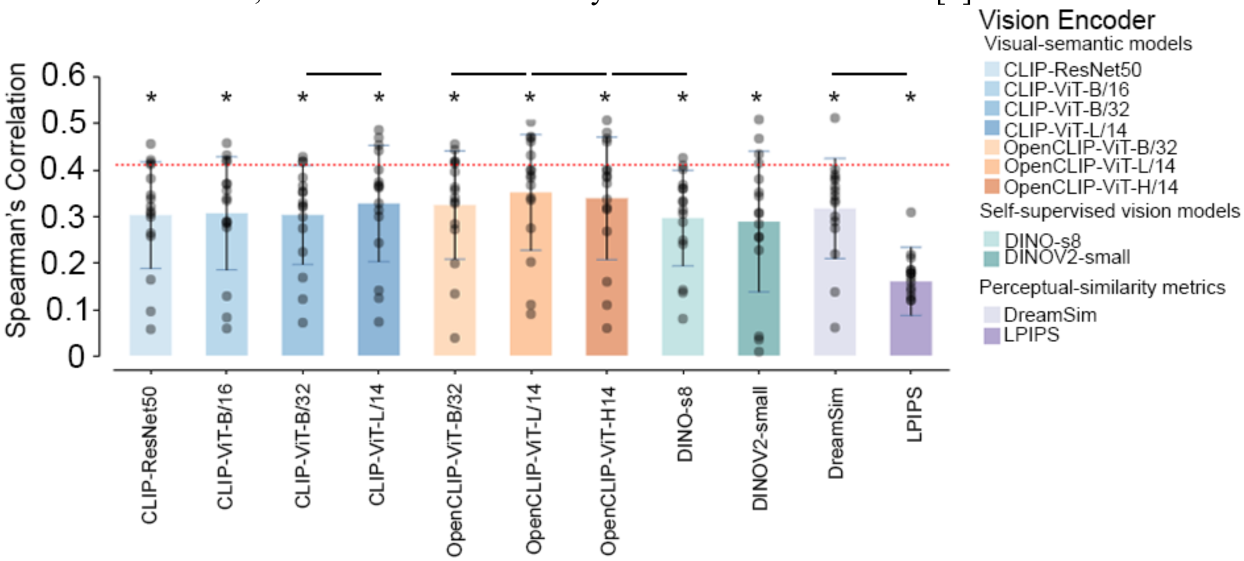

Fig S.6. Spearman's correlation between an individual's Vision RDM and Image-feature RDM from each tested vision encoder. The bars represent the average correlations across participants. The block dots represent the individual participants. The red dotted line indicates the lower bounds of the noise ceiling of human visual judgment results. On top of each bar, * indicates $p < 0.005$ for model-specific one-sided signed-rank tests against zero. The horizontal black bar indicates $p<0.05$ for two-sided pairwise signed-rank tests between two nearby vision encoder models shown in the plot.

Space of Morphable Material Appearance. Given two images of a pair of source and target materials, we can produce a morphing sequence that smoothly transforms the material appearance in the image space. With a pair of source and target generators, G_{source} and G_{target}, we apply linear interpolation to the models' weights at all convolution layers, while also interpolating between the latent codes drawn from the corresponding latent spaces W_{source} and W_{target} (see Method in main paper). We can synthesize the corresponding morphed material at any interpolation step, λ.

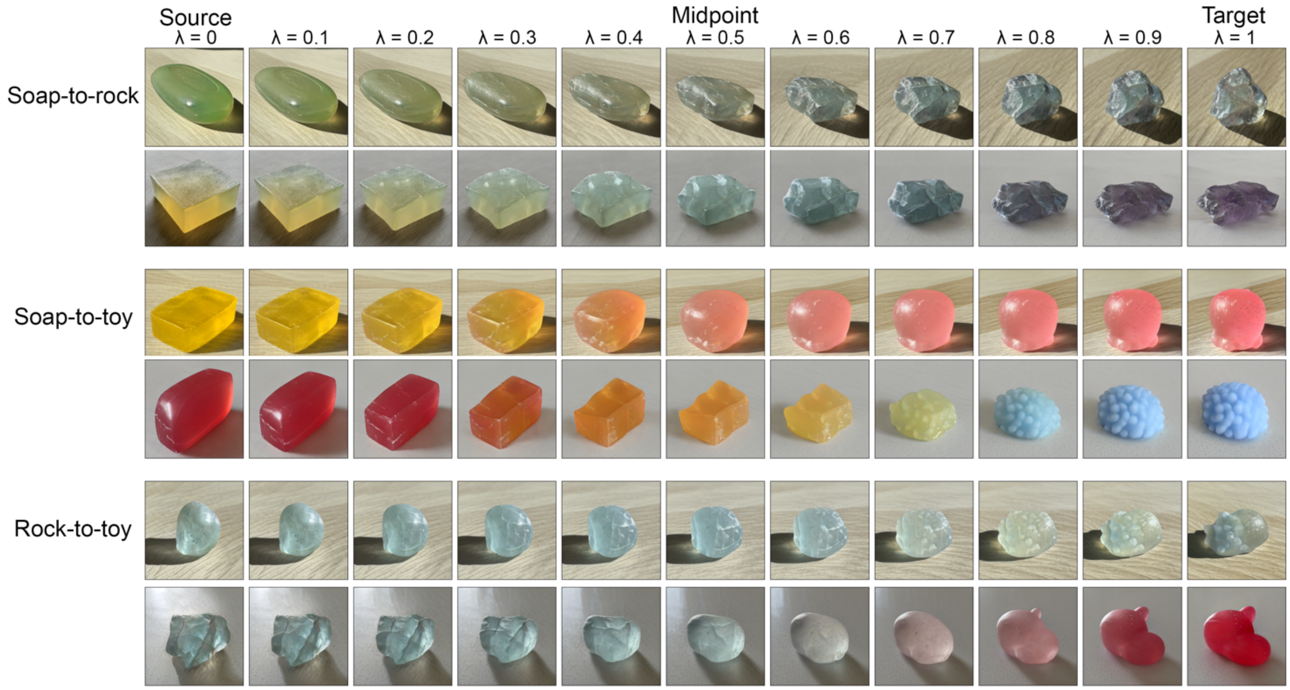

Fig S.7. Cross-material morphing examples. The source material transforms into the target material with a nine-step interpolation.

Interactive plot of Annotated MDS of the group average Vision RDM

In Supplementary Material **S1. File**, we provide the annotated MDS with the aggregated human text description of each material aspect, “colorfulness”, “optical properties”, “surface texture”, “mechanical properties”, and “material name.”

Size of the latent feature vectors from the pre-trained vision and LLM model

Table S.1. Extracted feature vector size of vision and LLM models.

Vision Model Name	Feature Vector Size
OpenCLIP-ViT-B/32	512
OpenCLIP-ViT-L/14	768
OpenCLIP-ViT-H/14	1024
CLIP-ViT-ResNet50	1024
CLIP-ViT-B/16	512
CLIP-ViT-B/32	512
CLIP-ViT-L/14	768
DINO-s8	384
DINOV2-small	384

LLM Model Name	Feature Vector Size
CLIP-ViT-B/32	512
Sentence-BERT	384
GPT-2	768
OpenAI Embedding V3-small	1536

References

[1] Oquab, Maxime, Timothée Darcet, Théo Moutakanni, Huy Vo, Marc Szafraniec, Vasil Khalidov, Pierre Fernandez et al. "Dinov2: Learning robust visual features without supervision." arXiv preprint arXiv:2304.07193 (2023).
[2] Fu, Stephanie, Netanel Tamir, Shobhita Sundaram, Lucy Chai, Richard Zhang, Tali Dekel, and Phillip Isola. "Dreamsim: Learning new dimensions of human visual similarity using synthetic data." arXiv preprint arXiv:2306.09344 (2023).
