# Supplementary figures and images for: Probing the link between vision and language in material perception using psychophysics and unsupervised learning
Source: PLoS Comput Biol. 2024 Oct 3;20(10):e1012481. doi: 10.1371/journal.pcbi.1012481 (PMC11478833; doi:10.1371/journal.pcbi.1012481)

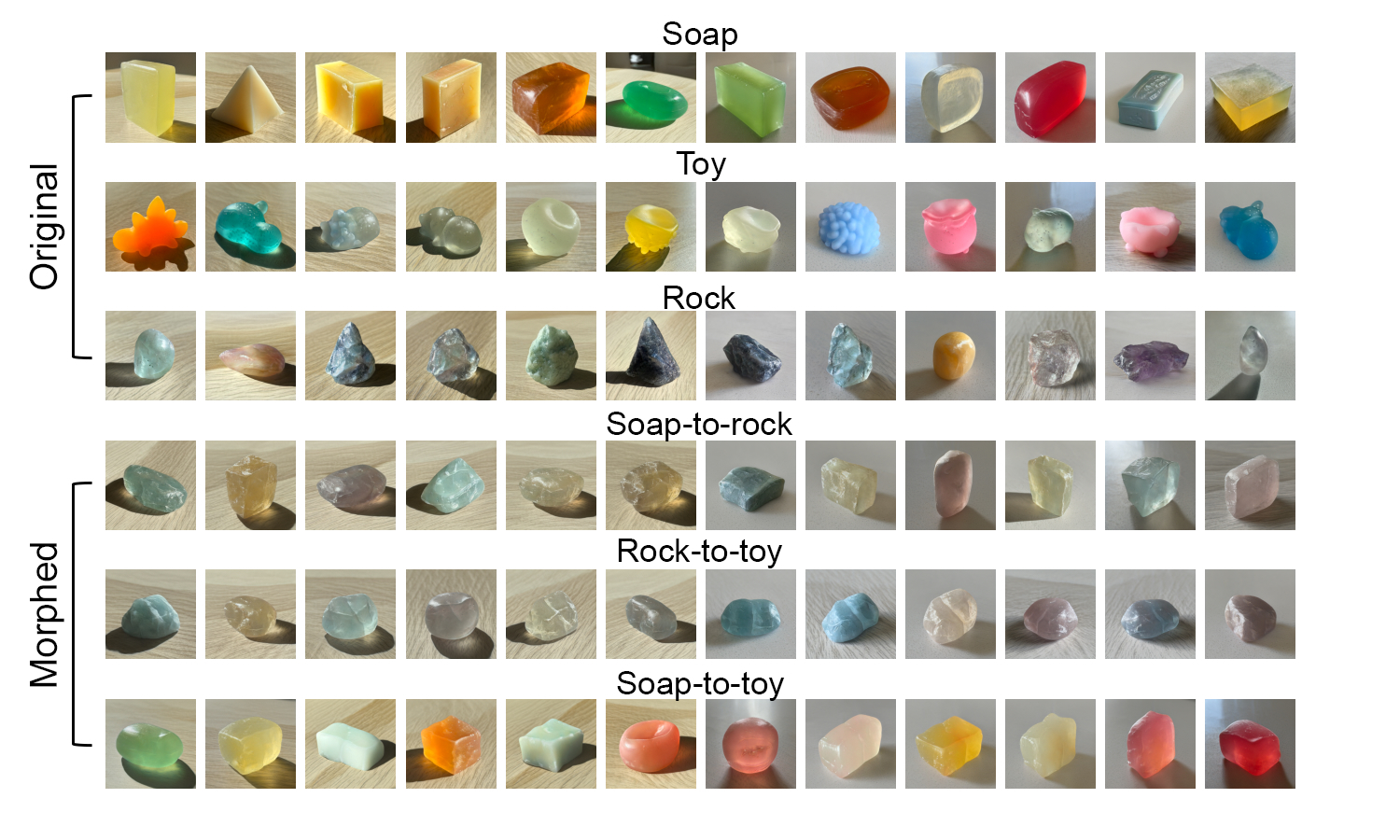

Supplement: S1 Fig — (TIF) [file pcbi.1012481.s002.tif]

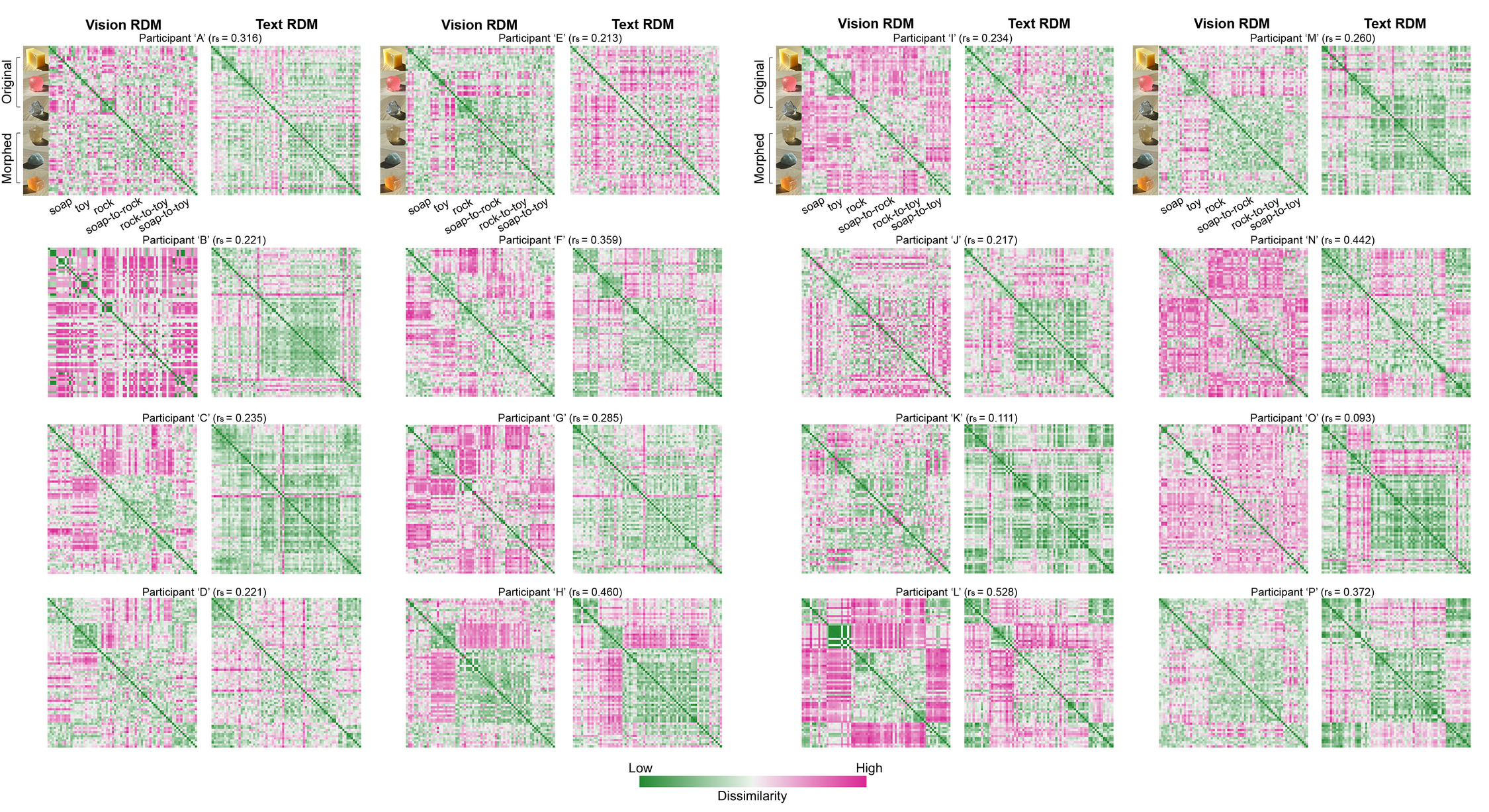

Supplement: S2 Fig — The Text RDMs are based on the CLIP’s text embedding results, as illustrated in the main paper Fig 4A. The Spearman’s correlation (rs) between the participant’s own Vision and Text RDMs is marked on top of each pair of RDMs. (TIF) [file pcbi.1012481.s003.tif]

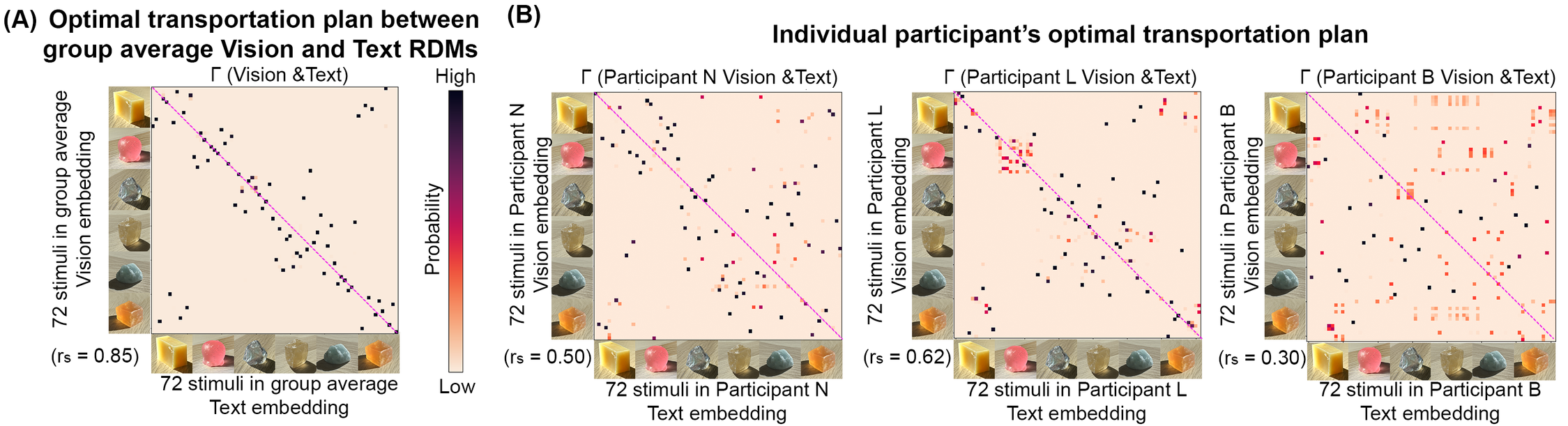

Supplement: S3 Fig — The Text RDMs are based on OpenAI Embedding V3-small. (A) Optimal transportation plan matrix (Γ) between group average Vision and Text RDMs. (B) Optimal transportation plan matrix of individual participant’s Vision and Text RDMs. The Spearman’s correlation (rs) between the Vision and Text RDMs is noted in the bottom left corner of the Γ matrix. (TIF) [file pcbi.1012481.s004.tif]

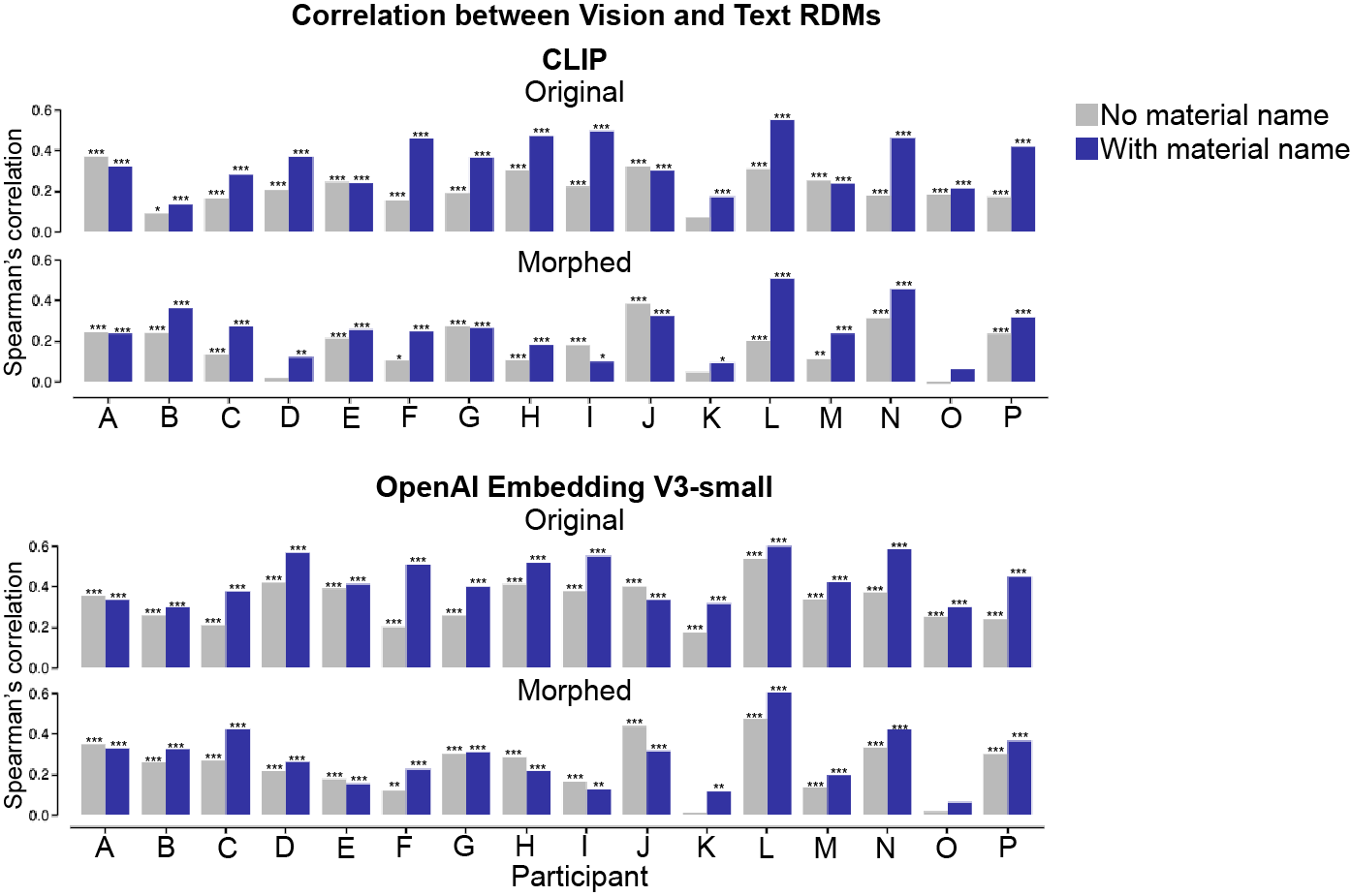

Supplement: S4 Fig — Top: text embedding derived from CLIP’s text encoder. Bottom: text embedding derived from OpenAI Embedding V3-small. The blue bars indicate Spearman’s correlation values when all text features are included to construct the Text RDM. The gray bars indicate the correlation values when the “material name” is excluded from constructing the Text RDM. Asterisks indicate FDR-corrected p-values: *** p < 0.001, ** p < 0.01, and * p < 0.05. (TIF) [file pcbi.1012481.s005.tif]

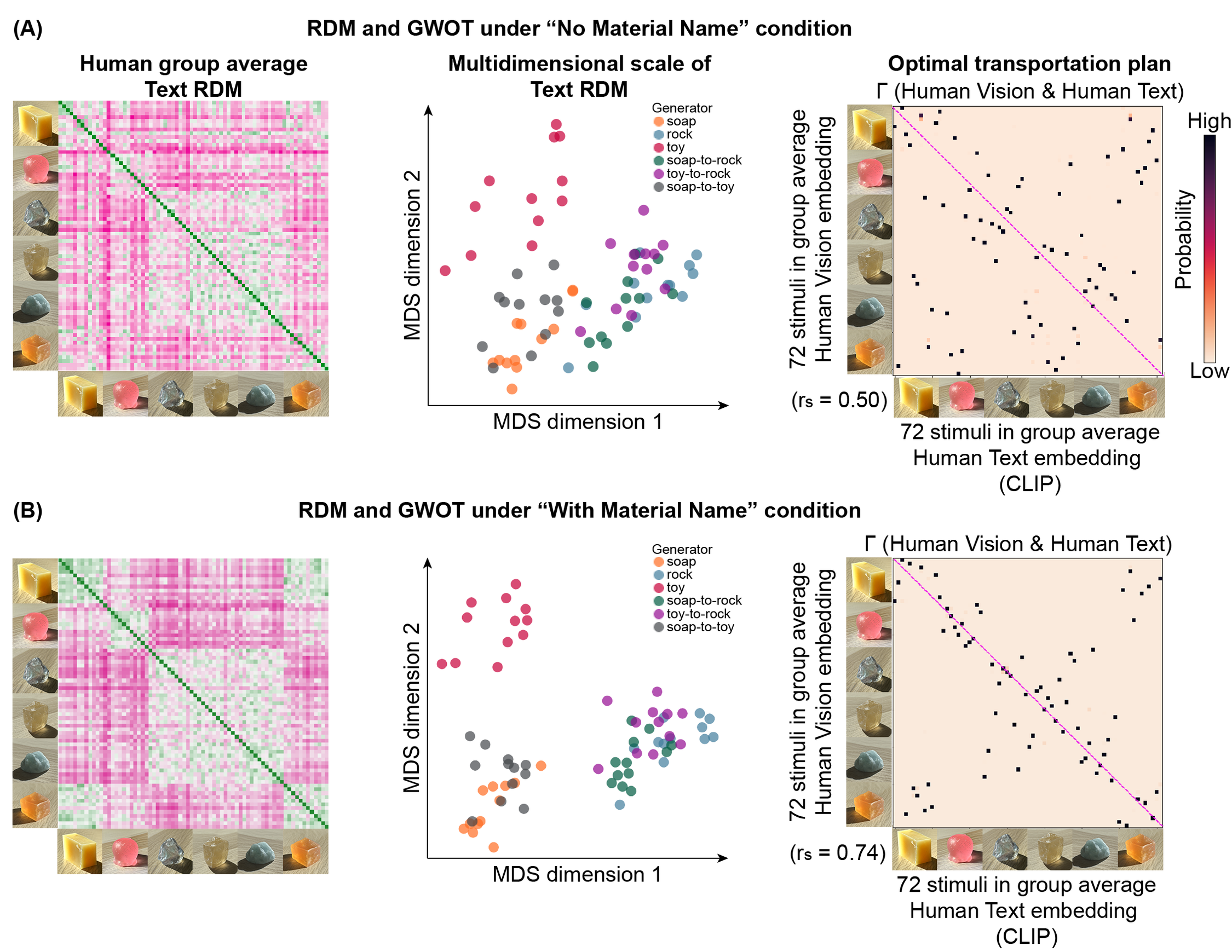

Supplement: S5 Fig — Under each text embedding condition, the leftmost column shows the group average Text RDMs. The middle column is the MDS embeddings of the group average Text RDMs. The rightmost column shows the optimal transportation plans that compare the group average human Vision RDM (from the Multiple Arrangement task) with the group average Text RDM. (A) “No Material Name” (when the “material name” is removed from the text embedding) (B) “With Material Name” condition. (TIF) [file pcbi.1012481.s006.tif]

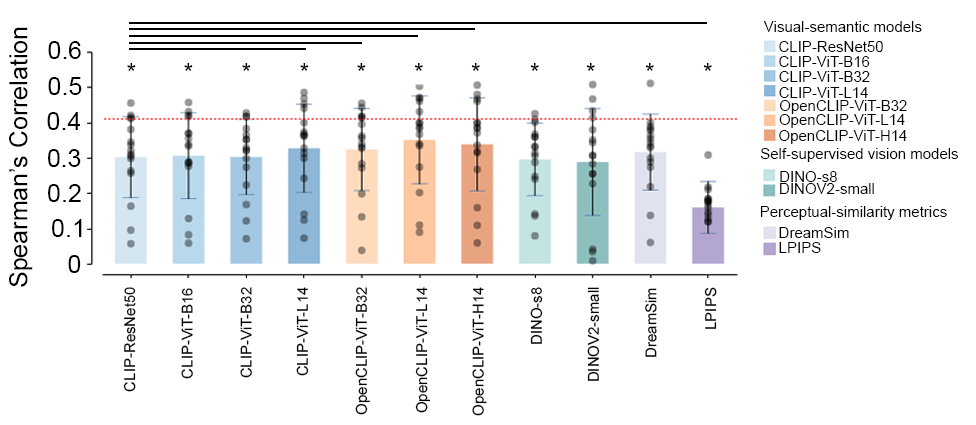

Supplement: S6 Fig — The bars represent the average correlations across participants. The block dots represent the individual participants. The red dotted line indicates the lower bounds of the noise ceiling of human visual judgment results. On top of each bar, * indicates p < 0.005 for model-specific one-sided signed-rank tests against zero. The horizontal black bar indicates p < 0.05 for two-sided pairwise signed-rank tests between two nearby vision encoder models shown in the plot. (TIF) [file pcbi.1012481.s007.tif]

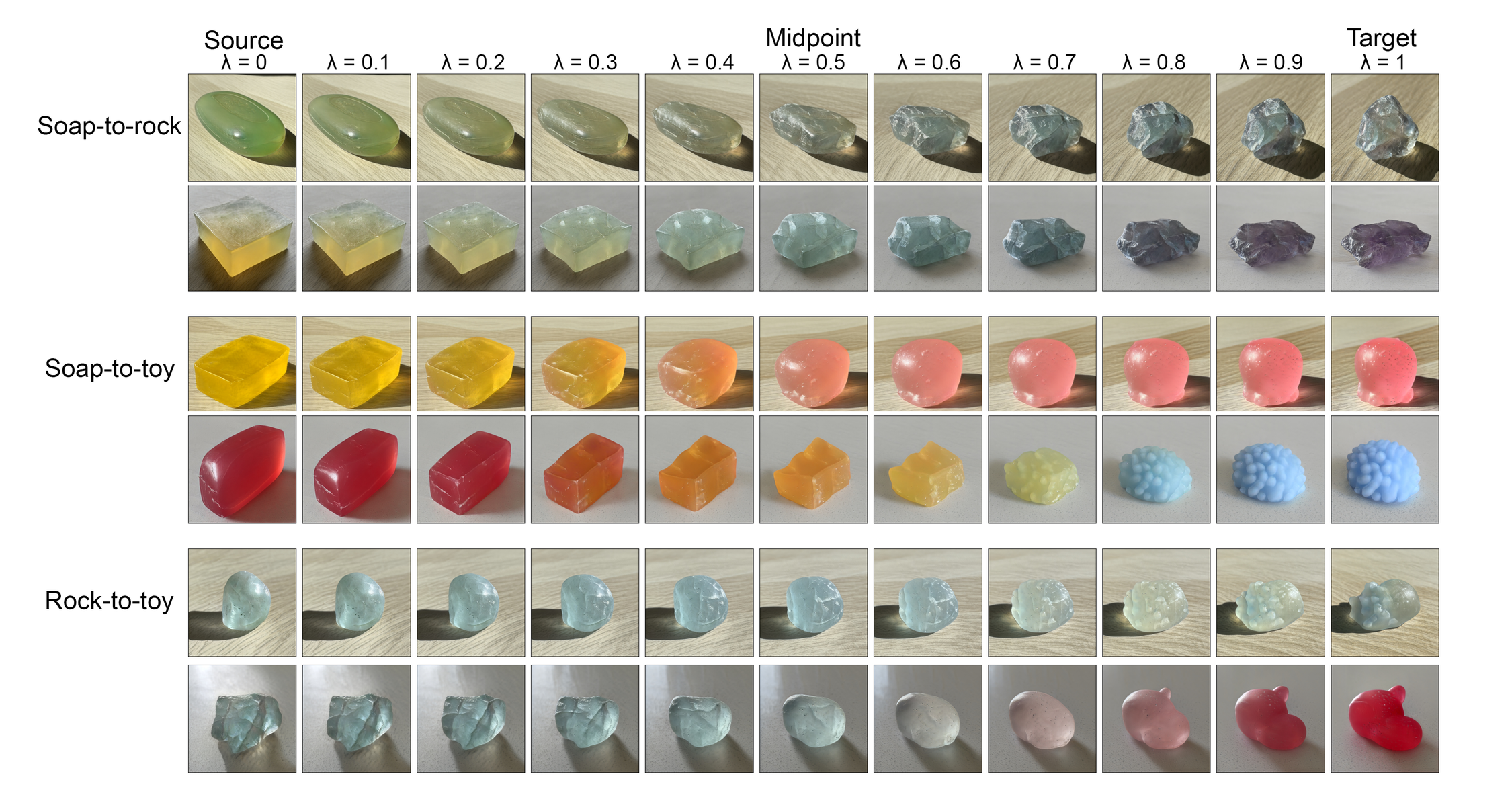

Supplement: S7 Fig — The source material transforms into the target material with a nine-step interpolation. (TIF) [file pcbi.1012481.s008.tif]

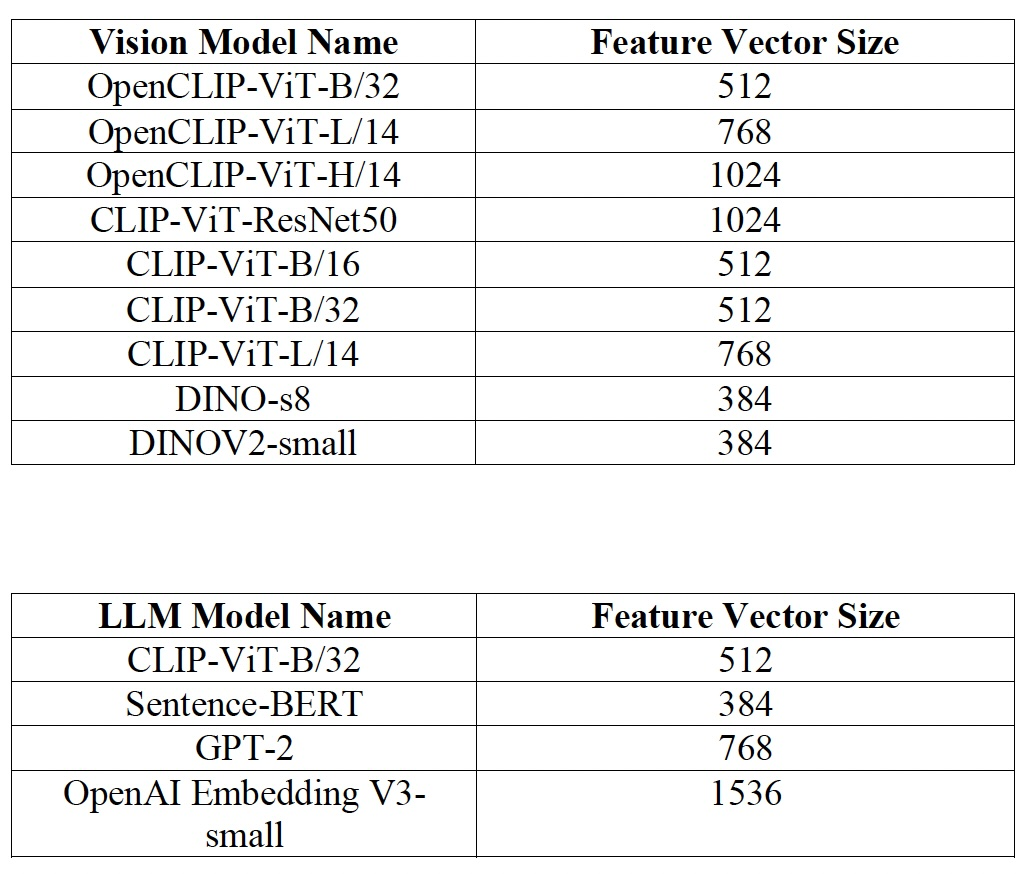

Supplement: S1 Table — (TIF) [file pcbi.1012481.s009.tif]
