# Supplementary material for: Probing the link between vision and language in material perception using psychophysics and unsupervised learning
Source: PLoS Comput Biol. 2024 Oct 3;20(10):e1012481. doi: 10.1371/journal.pcbi.1012481 (PMC11478833; doi:10.1371/journal.pcbi.1012481)
Supplement: Attachment — Submitted filename: Response to Reviewers.pdf [file pcbi.1012481.s011.pdf]

Dear Editor and Reviewers,

Thank you so much for your valuable feedback! We addressed your concerns and edited our manuscript based on your suggestions.

We provided a point-by-point response to each of the reviewers. Our responses are in blue text.

Meanwhile, we made the following major revisions to our main manuscript and the Supplementary Material.

- We added texts in the manuscript based on the reviewers' comments and marked the changes in red in the revised main manuscript.
- We performed additional analysis and added the figures in the updated Supplementary Material (Fig S.4, Fig S.5, File S.1, and Table S.1).
- We reordered and numbered the figures and tables in our Supplementary Material.

Once again, thank you very much for your time and efforts!

Sincerely

Chenxi Liao,

On behalf of all authors

Response to Reviewers

Reviewer #1: This paper presents a study about material appearance perception, comparing representations found by deep neural networks and human annotations for two different modalities: vision and language. For vision, the authors use image-based representations found by OpenCLIP and DINO models, and human visual similarity judgements (by placing images in a two-dimensional space); on the other hand, for language, they use semantic representations from various large language models (including OpenCLIP, but also Sentence-BERT or GPT2) and human descriptions of materials, in the form of natural language words. The objective is to analyze the vision-language relationship in the context of material perception, concluding that (within the conditions of their study), human visual judgements of material appearance correlate better with representations found by models that combine vision and language representations than with purely vision-based models. This conclusion, together with other interesting findings (e.g., highly lower correlations when using material names vs. removing them) is useful to design better computational models that mimic human visual perception.

Human perception of materials and how to model it with learning-based approaches is an open topic of research, and with the advent of large vision-language generative models (i.e., GPT, CLIP, diffusion...), I find it very important to thoroughly analyze the internal representations of such models and their correlations to human behavior, instead of using them as "black boxes". I think this paper does a very good job in this line, with useful contributions.

The paper is very easy to read, well motivated, and with a solid methodology. I find the approach convincing, and their design decisions reasonable (see below my only concerns in this regard).

I include here a couple of concerns that I would like the authors to better clarify/discuss in the paper:

- “Regarding the dataset, I wonder why to limit their images to these specific three categories (soap, toy and rock) and their interpolations in the StyleGAN space. One could argue these images represent well a wide variety of material appearance (e.g., quite different optical and mechanical properties, including translucency), and the material morphing possibility could be very useful to analyze material properties "out of context". However, I would appreciate a better motivation of this selection of the data, and an explicit mention that their conclusions might be biased towards this particular space of appearance.”

  We selected the materials (soaps, rocks, and squishy toys) due to several considerations. First, we intentionally focused on the translucent materials. We want to extend our previous Translucent Image Dataset (Liao et al., 2023) so that the enlarged datasets could be a candidate real-photo dataset to study the perception of various optical properties, especially for translucency, transparency, glossiness, etc. Second, within a coarse material category (e.g., soaps), we want the samples to cover a range of material properties such as glossiness and translucency, surface texture, geometric complexity, and colors. Meanwhile, we want the selected material categories to have unique characteristics. For example, soaps and rocks are very different in their surface geometry and body color. Soaps and squishy toys are distinct in their shapes and surface smoothness. All three selected materials are also unique in their tactile and mechanical properties.

  We acknowledge that the current dataset of our material selection could be limited in its diversity. Our effort is a step towards building a more comprehensive large-scale real photo dataset of diverse materials. We added this limitation in the Discussion.

  We added the following in the Discussion section of the main manuscript, Line 390:
  “…Our current dataset focuses on translucent materials, which share certain visual characteristics. We acknowledge that the current dataset of our selection of material could be restricted in its diversity. Our effort is a step towards building a more comprehensive large-scale real photo dataset of diverse materials.”

- Regarding the textual descriptions gathered from participants, the authors say that participants can "freely" articulate their experience with "unrestricted" descriptions. However, I have understood that the participants have a pre-defined set of characteristics to describe (e.g., material name, color) and they only use words or small phrases (vs. full sentences), so it sounds a bit overstated. I see it as a reasonable design decision, but it could better motivated and explained in the paper. Vision-language models, such as CLIP or GPT, can also manage long and free full-sentence descriptions, and the conclusions of the study (e.g., that participants are more diverse in the visual task than in the language one) could be different in this case. It would be helpful if the authors could offer more insights about this decision, or consider it as future work.

  In a small-scale pilot experiment, we gave participants minimal restrictions and allowed them to describe the material in a completely free manner. Participants were asked to “describe the material in the image.” The participants reported that they had a hard time coming up with the aspects of the materials they would write about. Even in this complete free-form description, the words they came up with are quite limited. In the current experiment, we chose a subset of the material attributes, hoping to reduce the mental effort for participants to think of what to describe and seek to encourage them to describe each attribute more actively.

  In the reported experiments, we told the participants the following:
  “Please provide keywords or short sentences to describe the material from the following aspects: The Name of the material, Color, Optical Properties, Mechanical Properties, and Surface Texture.”

  We also encouraged the participants to describe in the way they wanted and assured them that the questions were not judged by correctness. Given that our stimuli only contain a single object in a simple scene, we believe the current method of collecting verbal descriptions is suitable for the experiment. In the future, when we investigate more complex scenes, we can consider the complete free form of description.

  We edited the Methods section of the main manuscript, Line 475:
  “In the experiment instruction, the participants were told, “Please provide keywords or short sentences to describe the material from the following aspects: The name of the material, color, optical properties, mechanical properties, and surface texture.” We also encouraged the participants to describe the material attributes in the way they wanted, and assured them that the task was subjective, without correct answers. ”

Also, a few questions and suggestions:

- The "Space of Morphable Material Appearance", up to what point is it "controllable"? I see great interpolation results, could we extrapolate also in this space?

  The current work demonstrates an interpolation method that supports image-to-image material morphing with good transformation smoothness. Although the interpolation step can be linearly controlled, the image-level difference between the interpolated points is not linear due to the non-linearity of StyleGAN’s layer-wise generative process.

  The extrapolation does not seem to lead to satisfactory results. When the step size is small (e.g., morphing step = 1.1), extrapolation from a source to a target material leads to further variation of the target material’s appearance, such as shape and color. The example figure below shows that the extrapolation from soap to a rock (row 1) does not make the target material more “rock-like.” When the step size increases, the output image has unnatural colors and more artifacts. This is possible because the extracted latent code and the layer-wise generator are less likely to match and produce reasonable output images.

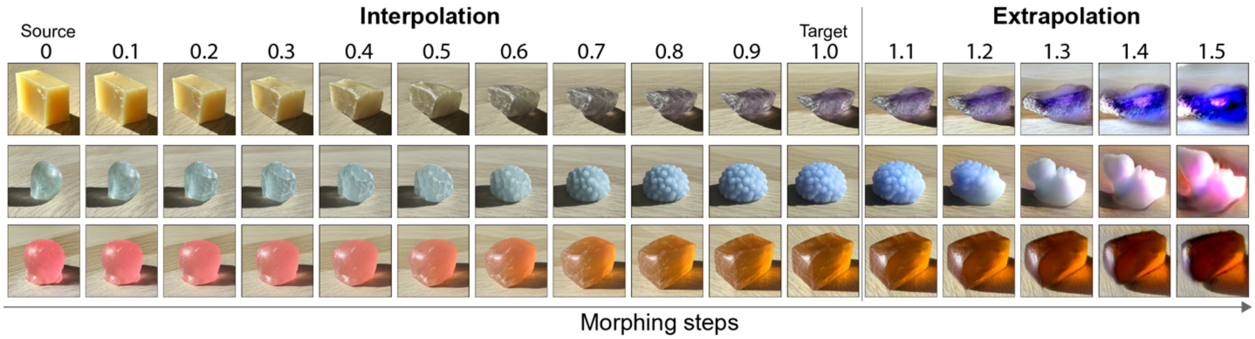

- I find the "removing material names" experiment and analysis very interesting. Does it also exhibit a significant change in correlation for *morphed* materials without a clear category? Have you run any additional analysis on that?

  Thank you for the suggestion.

  In short, Yes, removing “material name” also reduces correlation in “morphed” materials, similar to the “original” materials. Following your suggestion, we conducted an additional analysis comparing the Vision and Text RDMs (text embedding from CLIP or OpenAI Embedding V3-small) of the 36 images of ‘original’ (soap, rock, toy) and 36 images of morphed (soap-to-rock, soap-to-toy, toy-to-rock) materials. We added the following figure to the Supplementary Material of our main paper. When ‘material name’ is removed from the word embedding, we observed a significant decrease (at the confidence level of 95%) in vision-language correlation for both “original” and “morphed” material conditions (Wilcoxon one-sided signed-rank test, CLIP: original material $W(16) = 124$, $p = 0.001$, morphed material $W(16) = 119$, $p = 0.003$; OpenAI Embedding V3-small: original material $W(16) = 129$, $p = 0.0002$, morphed material $W(16) = 103$, $p = 0.037$).

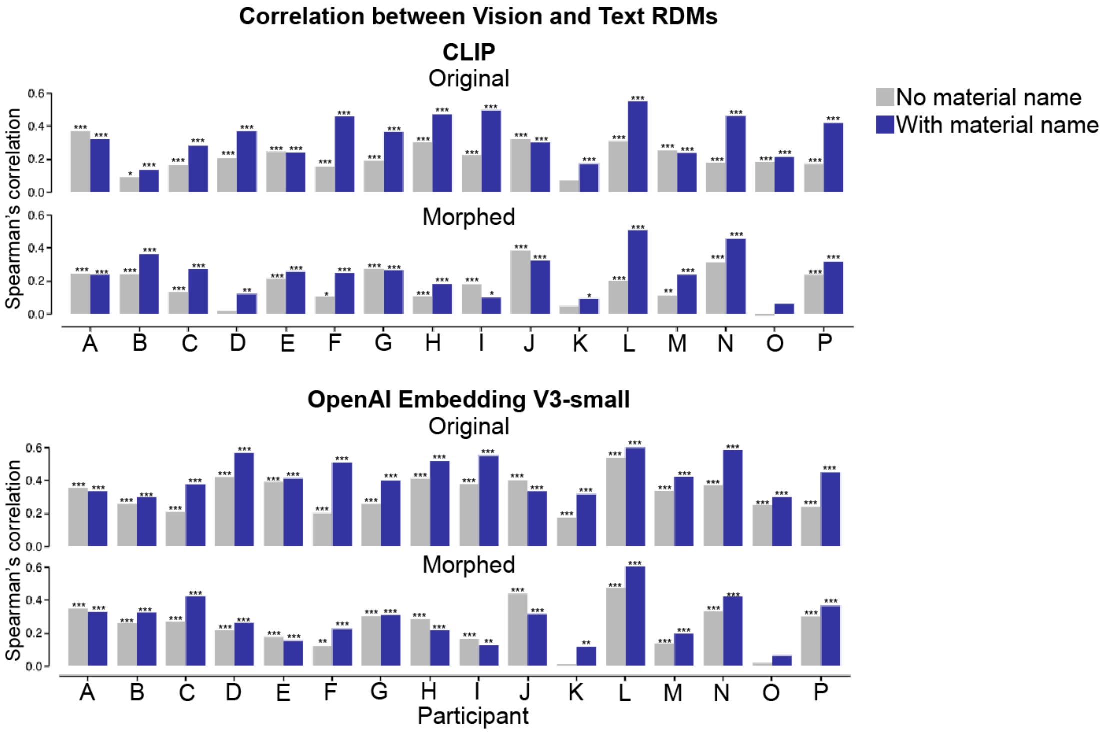

Fig S.4. Correlation between Vision and Text RDMs with ROI for the original “original” (soap, rock, toy) and “morphed” (soap-to-rock, soap-to-toy, toy-to-rock) materials. Top: text embedding derived from CLIP’s text encoder. Bottom: text embedding derived from OpenAI Embedding V3-small. The blue bars indicate Spearman’s correlation values when all text features are included to construct the Text RDM. The gray bars indicate the correlation values when the “material name” is excluded from constructing the Text RDM. Asterisks indicate FDR-corrected p-values: *** $p<0.001$, ** $p<0.01$, and * $p<0.05$.

We added the following in the Result section of the main manuscript, Line 194:

“ We observed a similar effect when separately comparing the perceptual RDMs of the 36 images of “original” and 36 images of the “morphed” materials. Removing “Material Name” also reduces vision-language correlation in “morphed” materials (see Supplementary Material S4 Fig). ”

- Did you consider including in the analysis the representations from a neural network trained *supervisedly* for a material similarity task?

Thank you for this suggestion. We did not train a neural network with supervised learning as it requires many trials of human similarity judgment that are beyond the scope of the current manuscript. In the future, we will consider building a supervised model using large-scale human perceptual data.

Typos / minor references corrections:
- l.86 behavior*al* tasks
- References consistency: et al. vs. list of authors
- Reimers et al. Sentence-BERT *EMNLP 2019*
- Deschaintre et al. The visual language of fabrics *ACM Trans. on Graphics 2023*
- Serrano et al. An intuitive color space... *ACM Trans. on Graphics 2016*

Thank you for pointing these out. We corrected all the typos and the format of references in the main manuscript.

Reviewer #2: The project takes the excellent Soap-trained generative model from the authors' 2023 paper, and extends it by fine-training on additional photos in order to be able to generate images of Soap, Rock, "Squishy toys" (soft rubber/plastic/silicone), or any morph between these three categories, with variable lighting, colour, and background surface.

The work is very technically proficient, and the visual quality of the generated objects is exceptionally good. The continuous "Space of Morphable Materials" is an excellent stimulus-generation tool that has only in the past couple of years become technically possible. The creation of abstract object feature spaces that can be smoothly traversed, with visually photorealistic images at each point, but no physically-defined ground-truth, opens up lots of possibilities for probing perception.

Having generated a diverse set of 72 ambiguous-material stimuli, the authors then collect two sets of behavioural data: arrangements on a 2D screen in terms of visual similarity, and verbal descriptions which are input to a Large Language Model (of various types) in order to produce a measure of verbal similarity. These human-produced visual and verbal similarities are then compared in various ways: how well do visual and verbal similarities correspond within and across participants, as quantified by RDM correlation or an optimal transport algorithm? How well can visual features extracted by other DNNs predict the human visual similarity judgements? How about combining verbal similarities and DNN-computed image features to predict human visual similarities?

In each case the answer is broadly that the different similarity spaces correspond moderately well with one another, but don't explain all the non-noise variance. Figures 4B and 5 provide a nice intuition for the human data - in both visual and text-derived similarities, people create three broad categories, which overlap partially with object identity/generative model, and somewhat better with the perceived mechanical properties, preferred material name, and hue/saturation.

The main take-away message is that there's a fair bit of overlap between statistical image features, material properties, human-perceived visual similarity, and human-described material similarity (at least within this constrained material sub-space), but that verbal descriptions can't fully capture the image-to-image nuances we perceive.

These seem like unsurprising conclusions, but the project is a beautifully illustrated technical tour-de-force, and may serve as an inspiring proof-of-principle for a method to generate stimuli that may soon be widespread in psychology experiments.

MINOR COMMENTS

- It would be good to cite the very small number of other papers that have so far applied unsupervised learning to the study of material perception:

Metzger, A., & Toscani, M. (2022). Unsupervised learning of haptic material properties. _Elife_, _11_, e64876.

Storrs, K. R., Anderson, B. L., & Fleming, R. W. (2021). Unsupervised learning predicts human perception and misperception of gloss. _Nature Human Behaviour_, _5_(10), 1402-1417.

Thank you for your feedback. We accidentally deleted the citations in the previous submission. We have now added them to the manuscript.

We added the following sentence in the Introduction of the main manuscript, Line 50:

“Without requiring annotated data, unsupervised learning has been recently applied to the study of human material perception [Storrs et al., 2021; Metzger & Toscani, 2022; Liao et al., 2023].”

Reviewer #3: The authors investigate the link between visual judgments and verbal descriptions of material qualities. In a visual task they ask participants to arrange images of materials according to similarity. In a subsequent verbal task participants had to complete a verbal statement about materials qualities by filling in the blanks in a preformulated sentence. Data from both tasks was converted into Representational Dissimilarity Matrices and correlation as well as optimal transport between these RDMs assessed. At the individual level the correlation and optimal transport between visual and verbal RDMs were moderate, but increased at the group level. Deviations from ideal in the optimal transportation plan matrix were most frequently for within category stimuli and for mix-category stimuli. The authors conclude that visual and verbal representation align only coarsely, at the categorical level. And, that visual representations of materials are richer than verbalized semantic features.

Materials and their qualities play a huge role in everyday life, shaping our interactions with objects. Despite this important we still know very little material perception works, including how the brain represents materials. Therefore, to what extend verbal and visual representations of materials have a similar representational structure is a very interesting and important one. The authors use deep learning in combination with behavioral experiments to address this question. The proposed methods and modeling could prove useful beyond the area of material perception.

I have a few comments and questions that might help revising the manuscript.

Stimuli:
The stimuli were images of objects with different material qualities: soap, rock and squishy toy, as well as mixed version between these. Images were generated with a generative adversarial network that was first trained on real photographs of soap pieces and then subsequently on the other two categories. Mixed category stimuli were created by interpolating between layer-wise latent codes of respective trained GANS.

• Why were just 3 categories used, and why these particular ones? Looking at Figure 2 the differences between the material categories seems to be mostly shape related – optically, there seems to be quite a bit of overlap (even for the non-morphs, e.g. translucent 'rocks').

Thank you for your feedback. Although there are only three coarse categories of materials, the dataset's material appearances are diverse.

We selected the three materials (soaps, rocks, and squishy toys) due to several considerations. First, we intentionally focused on the translucent materials. We want to extend our previous Translucent Image Dataset (Liao et al., 2023) so that the enlarged datasets could be a candidate real-photo dataset to study the perception of various optical properties, especially for translucency, transparency, glossiness, etc. Second, within a coarse material category (e.g., soaps), we want the samples to cover a range of material properties such as glossiness and translucency, surface texture, geometric complexity, and colors. Meanwhile, we want the selected material categories to have unique sets of characteristics. For example, soaps and rocks are very different in their surface geometry and body color. Soaps and squishy toys are different in their shapes and surface smoothness. All three selected materials are also unique in their tactile and mechanical properties.

As our image dataset focuses more on translucent materials, we intentionally choose materials that share the translucent material features. In the Supplementary Material S1 Fig, we provided more examples of the material appearances generated by our model. Our model could produce optically diverse material appearances, including opaque and moderately translucent ones.

• What if the stimulus set was extended to one very different category (say wood or clay)

It is possible to apply our method to generate images of very different materials (e.g., wood). In general, successful transfer learning benefits from the features/knowledge from pre-trained datasets. Given our success in using transfer learning to fine-tune StyleGAN in various image domains, it is possible to

extend our framework to other material categories with additional training data. Our future work will investigate the possibility and effectiveness of learning to generate images of even more diverse materials.

• Continuing the same argument: the main distinction between the three categories seems to be shape (not optical properties). Objects in all three categories are somewhat translucent. They differ, however in shape (it seems): e.g. soap is very regular, geometrically shaped (e.g. like platonic solids); rocks are irregular and jagged, and squishy toys all seem rounded. It would have been interesting to add this 3Dshape aspect as an answer option to the verbal description and to check how much this factor contributes to visual similarity judgements.

Thank you for the suggestion. Given the time constraint to redo the experiment, we would consider adding the description of “Shape” in future studies.

Procedure:
• All participants first completed the Multiple Arrangement task, and then the Verbal Description task.
– could this cause an order effect? Why not counterbalancing the order?

Our rationale for completing the Multiple Arrangement task first is to reduce the potential bias of our predefined set of material attributes (used in the Description Task) in visual judgments of materials.

To examine the ordering effect, all the experiments must be re-done within subjects with a larger scale of data collection (to also consider individual differences), and this will not be feasible within the scope of this current manuscript. In our pilot experiment, two participants performed experiments in the reverse order: Verbal Description first and then Multiple Arrangement. We also observed moderate-to-low vision-language correlation when we examined their Vision and Text RDMs, which is consistent with our current results. These pilot results suggest counterbalancing the ordering might not have a significant effect on our results.

• Verbal responses: ‘We used a fixed template to concatenate the five aspects that participants described an image: “It is a material of [material name] with the color of [color], it is [optical properties], it is [mechanical properties], and it is [surface texture].”
-In a sense this template forces the participants’ responses to a certain level of coarseness. Essentially the template encouraged participants to fill in the blanks with single or multiple adjectives. However, one can describe the look and potential feel of materials stylistically much more diverse than just listing a bunch of adjectives. I can imagine that this format was used because it is more suitable to use with LLMs, however it should be acknowledged that the potential complexity of the verbal response was restricted and – that this might have shaped the findings, e.g. ‘the persistent gap in using words to capture the nuanced visual differences among diverse material samples’, or the higher inter-participant correlation in the verbal task.
-In general, I think what is communicable about an image/material certainly depends on the linguistic skill of the communicator

Thank you for pointing out our unclear description. In the experiment, we did not use a template sentence but simply asked observers to describe the object from 5 material attributes freely. The template is only used to construct the text embedding using the participant’s response. We have clarified this procedure in Fig.3B and Methods section Line 485 in the main manuscript on how the template was constructed based on participants’ responses. We believe this experiment design could encourage them to actively consider the material attributes. Also, given our stimuli only contain a single object against a simple scene, we believe this will not restrict the complexity of verbal responses. We will consider more free-form expression when we create stimuli of more complex scenes.

We agree with the reviewer that the material description might depend on the participant's linguistic skills, which might be reflected in the individual differences we observed in our paper. We also acknowledge that when we use more complex stimuli, it would be important to use a completely free form of expression to examine the effect of individual differences in linguistic skills on vision-language correlation. For the current experiment, though, we believe the current form of collecting verbal responses is suitable.

We added the following to the Discussion section of the main manuscript, Line 385:

"In our current Verbal Description experiment, we provided a predetermined list of material attributes to encourage the participants to actively consider the material attributes. Given that our stimuli only contain a single object in a simple scene, the design is suitable for collecting verbal communication of materials. In the future, we will consider more free-form, even complete free-form expression when we create stimuli of more complex scenes. Our current dataset focuses on translucent materials, which share certain visual characteristics. We acknowledge that the current dataset of our selection of material could be restricted in its diversity. Our effort is a step towards building a more comprehensive large-scale real photo dataset of diverse materials."

• Figure 5: Displays the most frequently used word for each image aggregated across all participants. -Was there a cut off for minimum frequency? Suppose if all but two participants used a different label, than that label (used by 2 participants) would be the most frequent, even though it was not used very often overall.

No, we did not apply a cut-off for minimum frequency. The word frequency is determined on a stimulus-based basis. For each stimulus with a given attribute (e.g., material name), we aggregated the verbal descriptions from all participants and then counted the word occurrences. The word "frequency" could reflect participants' overall tendency to use certain words to describe a particular material attribute.

We added Supplementary Material S1. File, the interactive version of plots in Fig.5 of our main manuscript. The reader of interest could find the most frequent word along with its frequency for each stimulus. We provide the annotated MDS with the aggregated human text description of each material aspect, "colorfulness" (MA_annotated_color.html), "optical properties" (MA_annotated_optical.html), "surface texture" (MA_annotated_texture.html), "mechanical properties" (MA_annotated_mechanical.html), and "material name" (MA_annotated_naming.html).

We added the following to the caption of Fig.5:
"An interactive version of this plot is provided in the Supplementary Material S1 File."

• In the verbal task, do the authors think that being asked to provide a material name might bias/limit the subsequent description of the attributes? E.g. if I label this rock then it must be rough.

We think providing a material name might affect how participants judge certain mechanical material attributes, such as softness (e.g., when observers judge the object to be rock, they will often describe it as soft). As shown in Fig.5, the material name and softness are often correlated. On the other hand, the material names might not directly affect the description of optical properties (e.g., translucency, glossiness). However, in this experiment, we do not know the causal relationship between material name and material attribute. We will investigate this in the future.

• Figure 6 Participant B, who has the highest number of unique words also has lowest correlation between visual and verbal RDMs. Is a (relatively) high number of different words possibly "punished" by this analysis, because there will be less correspondence between any two stimuli (descriptions)?

The within-participant vision-language correlation could possibly be affected by the number of words the participant chose to describe each stimulus. Participants (e.g., Participant A, who used the highest number of unique words, Fig.6) whose verbal description includes larger variations may lead to more irregular text embedding and further reduce the vision-language correlation. Given our current data, it is difficult to say that a high number of unique words systematically leads to a low vision-language correlation.

• How comparable in terms of size are the feature vectors for visual and verbal representations? Perhaps this could be added to the methods.

Thank you for pointing this out. The visual representation from psychophysics does not have a feature vector; it is based on the RDM obtained from the Multiple Arrangement task.

The size of the image feature vectors varies depending on the pre-trained models. The visual feature vector sizes are relatively comparable with the text feature vectors from the LLMs.

We added the following table, Table S.1, to the Supplementary Material.

Table S.1. Extracted feature vector size of vision and LLM models.

**Vision Model Name**	**Feature Vector Size**
OpenCLIP-ViT-B/32	512
OpenCLIP-ViT-L/14	768
OpenCLIP-ViT-H/14	1024
CLIP-ViT-ResNet50	1024
CLIP-ViT-B/16	512
CLIP-ViT-B/32	512
CLIP-ViT-L/14	768
DINO-s8	384
DINOV2-small	384

**LLM Model Name**	**Feature Vector Size**
CLIP-ViT-B/32	512
Sentence-BERT	384
GPT-2	768
OpenAI's Embedding V3-small	1536

We also added the following sentence in the Methods section of the main manuscript, Line 531:

"The size of the image feature vectors varies depending on the pre-trained models (see Supplementary Material Table S.1)."

Discussion page Line 263 ff: ‘Our results show that when the material name is removed from the text embedding, the correlation between Vision and Text RDMs systematically decreases across participants (Figure 6). This may stem from the functional roles that nouns (material play in everyday language usage and highlights the significant role of material names in visual categorization. ‘

• This sounds a bit vague. Can the authors explain how removing the material name changes the representation/weights in the LLM? Can this be probed?

To clarify, we did not train the LLM on our collected text responses to obtain the embeddings. So, removing material names does not change the weights (parameters) of the LLM. To investigate the representation of the LLM, we conducted the MDS and GWOT analysis with the text embedding of the “No material name” condition (see our main manuscript Fig.6) derived from the CLIP’s text encoder. Our analyses suggest that removing the material name may further reduce the one-to-one mapping between verbal description and visual judgment of stimuli. We added the following Figure, Fig S.5, in the Supplementary Material. Similar to the “With material name” condition, we computed the Group Average Text RDM by taking the mean of the individual participants’ Text RDMs when the description of the material name is removed from the text feature embeddings (see Fig S.5A Leftmost panel). Hence, we applied the MDS to visualize the two-dimensional embedding of the “No material name” group average Text RDM. Compared with the MDS of the “With material name” (see Fig S.5B Middle panel), the data points in the “No material name” condition show less clear clustering (see Fig S.5A Middle panel). When comparing group average Vision RDM and the Text RDM with GWOT, removing the material name results in an optimal transport plan matrix Γ (see Fig S.5A Rightmost panel) that has an even smaller fraction of diagonal elements in its diagonal in comparison to the Γ from “With material name” condition (see Fig S.5B Rightmost panel). The top-1 matching rate also significantly dropped (top-1 matching rate, “With material name” condition = 12.5%, “No material name” = 1.38%).

We added this sentence in the Discussion, Line 325:

“We conducted the MDS and GWOT analysis with the human text embedding at the ‘No Material Name’ condition (see Supplementary Material S5 Fig). We observed that removing the material name may further reduce the one-to-one mapping between verbal description and visual judgment of material (Spearman’s correlation $r_s = 0.74$ for the ‘With Material Name’ condition to $r_s = 0.5$ for the ‘No Material Name’ condition).”

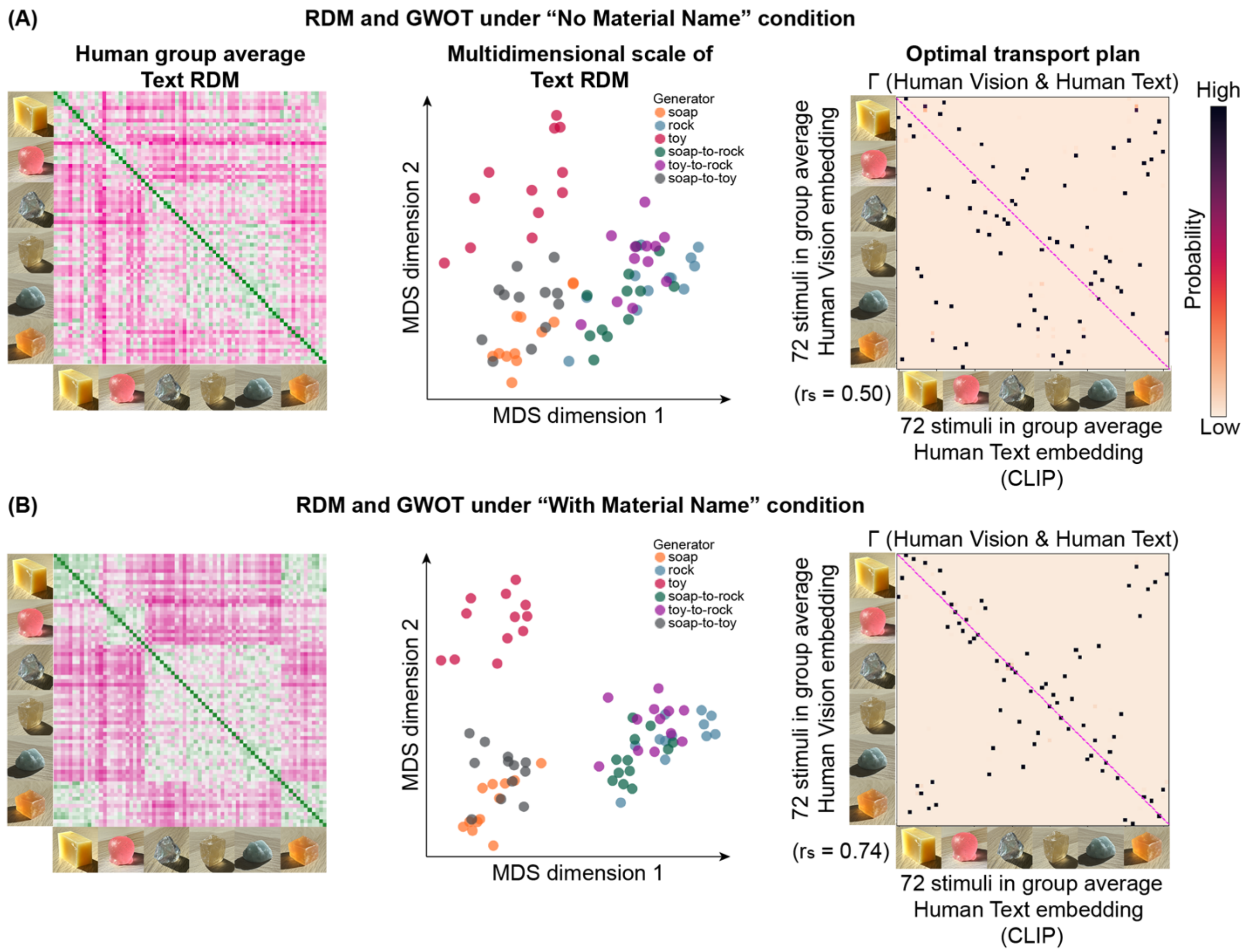

Fig S.5. Representational space of human text response in the “No Material Name” versus “With Material Name” condition based on CLIP’s text embedding. Under each text embedding condition, the leftmost column shows the group average Text RDMs. The middle column is the MDS embeddings of the group average Text RDMs. The rightmost column shows the optimal transport plans that compare the group average human Vision RDM (from the Multiple Arrangement task) with the group average Text RDM. (A) “No Material Name” (when the “material name” is removed from the text embedding) (B) “With Material Name” condition.
